# Associations of participation in community assets with health-related quality of life and healthcare usage: a cross-sectional study of older people in the community

**DOI:** 10.1136/bmjopen-2016-012374

**Published:** 2017-02-09

**Authors:** Luke A Munford, Mark Sidaway, Amy Blakemore, Matt Sutton, Pete Bower

**Affiliations:** 1 Manchester Centre for Health Economics, University of Manchester, Manchester, UK; 2 NIHR School for Primary Care Research, Manchester Academic Health Science Centre, University of Manchester, Manchester, UK; 3 Salford Royal NHS Foundation Trust, Salford, UK

**Keywords:** PUBLIC HEALTH, HEALTH ECONOMICS

## Abstract

**Background:**

Community assets are promoted as a way to improve quality of life and reduce healthcare usage. However, the quantitative impact of participation in community assets on these outcomes is not known.

**Methods:**

We examined the association between participation in community assets and health-related quality of life (HRQoL) (EuroQol-5D-5L) and healthcare usage in 3686 individuals aged ≥65 years. We estimated the unadjusted differences in EuroQol-5D-5L scores and healthcare usage between participants and non-participants in community assets and then used multivariate regression to examine scores adjusted for sociodemographic and limiting long-term health conditions. We derived the net benefits of participation using a range of threshold values for a quality-adjusted life year (QALY).

**Results:**

50% of individuals reported participation in community assets. Their EuroQol-5D-5L scores were 0.094 (95% CI 0.077 to 0.111) points higher than non-participants. Controlling for sociodemographic characteristics reduced this differential to 0.081 (95% CI 0.064 to 0.098). Further controlling for limiting long-term conditions reduced this effect to 0.039 (95% CI 0.025 to 0.052). Once we adjusted for sociodemographic and limiting long-term conditions, the reductions in healthcare usage and costs associated with community asset participation were not statistically significant. Based on a threshold value of £20 000 per QALY, the net benefits of participation in community assets were £763 (95% CI £478 to £1048) per participant per year.

**Conclusions:**

Participation in community assets is associated with substantially higher HRQoL but is not associated with lower healthcare costs. The social value of developing community assets is potentially substantial.

**Strengths and limitations of this study**

- This study addresses the lack of quantitative evidence on the effectiveness of community assets in improving the health of an older population.
- We use data on a large sample of individuals containing detailed information on community asset participation and health and healthcare usage.
- We apply a net-benefit societal approach to calculate the value associated with participation in community assets.
- Our ability to determine causation is limited by the cross-sectional data.

## Introduction

Policymakers across the world are becoming increasingly interested in improving health and well-being by creating a more inclusive community-based society. In 2010, the UK government stressed the need for a ‘Big Society’;^1^ one where individuals engage more with the facilities in their local community. The aim was to reduce inequalities and improve health and well-being, with a focus on localism, devolution, volunteerism and social enterprise.

One important aspect of the Big Society was ‘community assets’. A community asset is: ‘…the collective resources which individuals and communities have at their disposal, which protect against negative health outcomes and promote health status’ (Glasgow Centre for Population Health^2^). Examples of community assets include charity, voluntary or community groups. These community assets can lead to an increase in social inclusion and a decrease in loneliness, which have been associated with better health.^3–5^

Foot and Hopkins^6^ argued that community assets can achieve a number of goals, including (1) providing new ways of challenging health inequalities; (2) valuing resilience; (3) strengthening community networks and (4) recognising local expertise. McKnight and Kretzmann 7 introduced community asset mapping, which can be used to provide health promoters “with an understanding of how best to create the conditions required to maximise the potential for health” (ref. 8, p. 20). In later work,^9^ they introduced Asset Based Community Development, a system in which a community increases the health and well-being of its population using activities, skills and assets of (lower income) people and neighbourhoods. Mathie and Cunningham^10^ further developed this concept by stressing the importance of a sense of community belonging, social inclusion and social capital.

Reviews of the community asset literature in the USA^9–11^ and a limited amount of qualitative work have evaluated the effectiveness of community assets at improving health.^12^ The general consensus is that community assets improve the health of participants. However, there is limited quantitative evidence to support their effectiveness. A longitudinal study by Haslam *et al*^13^ showed that engagement with social groups, when compared with individual social engagement, significantly predicted improvements in subsequent cognitive function. This relationship became stronger with increasing age. Steffens *et al*^14^ showed that membership of social clubs around retirement age led to better quality of life and a reduced risk of premature death. Furthermore, two studies by Cruwys *et al*^15^ ^16^ have shown that membership of more clubs was associated with a lower probability of future depression and that identification with a social group predicts recovery from depression.

We aimed to contribute to the quantitative evidence by exploiting a cohort containing information on participation in community assets, health conditions and health-related quality of life (HRQoL). Further, we tested whether community assets and formal healthcare services are substitutes or complements. We used the results to calculate the net benefits of community assets.

## Methods

### Data

We used data from the National Institute of Health Research-funded Comprehensive Longitudinal Assessment of Salford Integrated Care (CLASSIC) study. CLASSIC is an evaluation framework designed to evaluate the Salford Integrated Care Programme (SICP). The SICP is a large-scale integrated care project to transform care for older people with long-term conditions and social care needs. The SICP aims to improve care via a number of mechanisms, including improved access to community assets.

**Figure 1:**
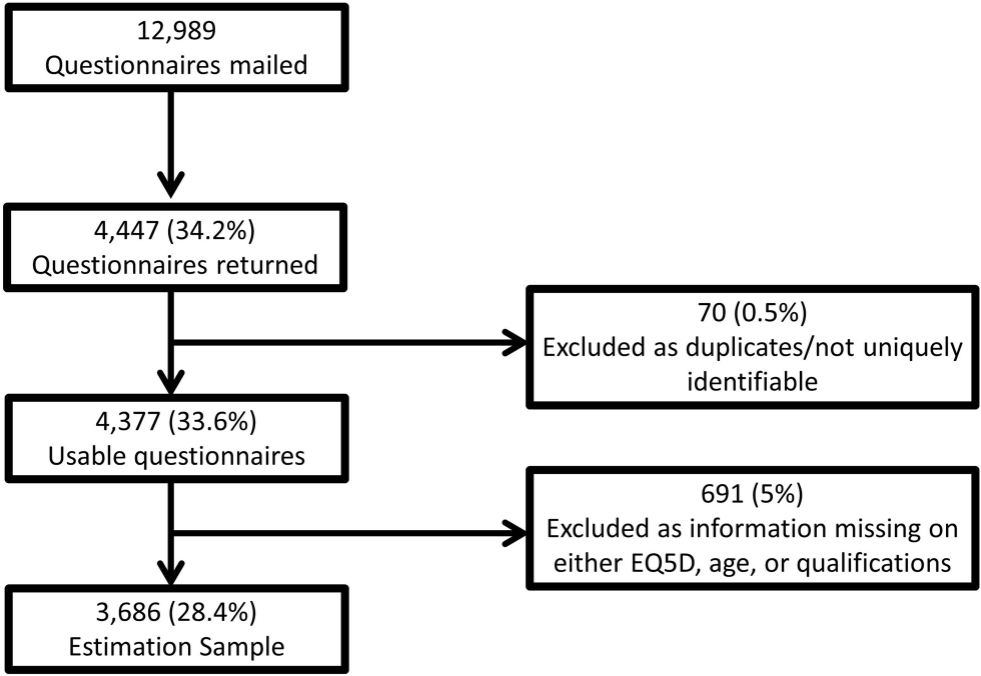
Flow chart explaining sample size.

Questionnaires were mailed to 12 989 individuals aged 65 years and older with at least one long-term health condition living in the Salford area (a city in the North West of England) between November 2014 and February 2015. These individuals were selected from the disease registers of 33 general practices. Responses were received from 4377 (34%) individuals. We analysed the 3686 (84%) respondents for whom complete data were available (figure 1).

### Variables

#### Health-related quality of life

We used the EuroQol-5D (EQ5D) to measure HRQoL.^17^ The EQ5D is the generic HRQoL measure of choice for the National Institute for Health and Care Excellence (NICE) in the UK.^18^ The EQ5D has five domains: ‘mobility’, ‘self-care’, ‘usual activities’, ‘pain/discomfort’ and ‘anxiety/depression’. We used the five-level version of the EQ5D (http://www.euroqol.org/eq-5d-products/eq-5d-5l.html). Individuals were asked to report their health today in each domain on a five-point ordinal score, where (1) corresponds to no problems; (2) slight problems; (3) moderate problems; (4) severe problems and (5) extreme problems/unable to do activities. To obtain the utility scores, we mapped the five-level version to the three-level version using the cross-walk tool described in van Hout *et al*.^19^

#### Usage of formal healthcare services

Each individual was asked four questions about their recent healthcare usage:

- ‘How many times have you seen the GP in the last 6 months?’
- ‘In the last 6 months, how often have you attended a hospital out-patient appointment?’
- ‘In the last 6 months, how often have you had to dial 999 and call an ambulance?’
- ‘In the last 6 months, how often have you attended an emergency department/casualty because of an emergency health problem?’

We analysed the numbers of general practitioner (GP) contacts and outpatient appointments separately, and then the combined costs of the four forms of healthcare usage in the last 6 months. We obtained the costs of outpatient attendances, ambulance callouts and casualty visits from National Health Service (NHS) reference costs.^20^ The cost of a GP visit was taken from the Personal Social Services Research Unit (PSSRU) Unit Costs publication.^21^ The costs at 2014/15 prices of each activity were: GP appointment £65.00; outpatient attendance £134.22; ambulance callout £96.35 and casualty visit £131.92.

#### Community asset participation

Individuals were asked ‘Have you attended or used any of the following community groups, activities and services in the last 6 months? (TICK ALL that apply)’ with possible responses: (1) trade unions; (2) group for the elderly or older people (eg, lunch club); (3) environmental groups; (4) youth groups (eg, Scouts, Guides and youth club); (5) parent–teacher association or school association; (6) Women's Institute or Townswomen's Guild or women's groups; (7) residents’ group or neighbourhood watch; (8) social club (including working men's clubs, rotary clubs); (9) education, arts, music or singing groups; (10) sports club, gym, exercise or dance groups; (11) religious group or church organisation; (12) other group or organisation and (13) charity, voluntary or community group.

An individual was classified as a community asset participant if they ticked yes to one or more of the above. The content of the list was based on the Health Survey for England^22^ and had been tested in another large study of older people in the same locality.^23^

#### Health conditions

Individuals were selected on the basis that they had at least one of the 23 following longstanding conditions according to their primary care health records: (1) asthma; (2) cancer (not including skin cancer); (3) chronic back pain or sciatica; (4) chronic bronchitis, chronic obstructive pulmonary disease (COPD) or emphysema; (5) chronic kidney disease; (6) colon problem, irritable bowel or colitis; (7) congestive heart failure; (8) depression, anxiety or emotional difficulties; (9) diabetes; (10) hard of hearing; (11) heart disease, angina, heart attack, bypass surgery or angioplasty; (12) high blood pressure; (13) high cholesterol; (14) osteoarthritis; (15) osteoporosis; (16) overweight; (17) poor circulation in legs; (18) rheumatoid arthritis; (19) rheumatic disease, fibromyalgia or lupus; (20) stomach problem, ulcer, gastritis or reflux; (21) stroke; (22) thyroid disorder; (23) vision problem and (24) other.

Individuals were asked to report whether or not they had each of the conditions using the Bayliss scale.^24^ Individuals were also asked how much each condition limited their daily activity on a five-point Likert scale (from (1) ‘not at all’ to (5) ‘a lot’). We defined the condition to be limiting if an individual ticked (4) or (5) and checked that the results were robust to including the middle category. We did not include depression because anxiety/depression is one of the domains of the EQ5D.

#### Demographic and socioeconomic characteristics

We controlled for gender and age using a series of 5-year age categories (ranging from 65–69 years, up to 85+ years). The reference age group is 65–69 years. We also controlled for living situation, coded as ‘live with spouse’, ‘live with other’ or the reference category ‘live alone’. We included binary variables for each of the following qualifications: ‘one or more Ordinary Level (O-Levels)/Certificate of Secondary Education (CSEs)/General Certificate of Secondary Education (GCSEs)’, ‘one or more A-Levels/AS-Levels’, ‘Degree’, ‘National Vocational Qualification (NVQ)’, ‘Trade qualifications’, ‘Professional qualifications’). An individual can tick multiple responses. The reference category was ‘no qualifications’.

### Statistical methods

We compared the mean EQ5D scores and levels of healthcare usage and costs between community asset participants and non-participants.

We then estimated three separate multivariate models for the EQ5D score, which included community asset participation and: (1) gender, age and socioeconomic characteristics only; (2) gender, age and socioeconomic characteristics and binary indicators of the presence of 23 long-term health conditions and (3) gender, age and socioeconomic characteristics and binary indicators of the presence of 23 long-term health conditions that limited activity (see above).

Following this, we estimated the same three model specifications for the number of GP visits, the number of outpatient attendances and the combined costs of four forms of healthcare usage over the last 6 months.

Finally, we combined the effects on health-related quality of life (HRQoL) and healthcare costs to produce estimates of the societal value of a year's participation in community assets using the net-benefit framework introduced by Stinnett and Mullahy.^25^ This involved multiplying each individual's EQ5D score by a value for a quality-adjusted life year (QALY) and subtracting the annual cost of their healthcare usage. Estimates of a ‘social value of a QALY’ have been estimated to be between £40 000 and £50 000.^26^ The threshold values for a QALY used by NICE in their decision-making on investment decisions are widely believed to be between £20 000 and £30 000. Claxton *et al*^27^ have recently questioned these values and instead recommend a threshold value of £12 936. We use a range of threshold values (£20 000, £30 000 and £12 936), and multiply our 6-month cost values by two to obtain annual estimates of costs. These measures of net benefit were then used as dependent variables in three further regression models, which included gender, age, socioeconomic characteristics and 23 indicators of the presence of long-term health conditions that limited daily activities.

## Results and discussion

### Participation in community assets

A total of 50% (1829/3686) of respondents reported participating in a community asset. Most participants reported participating in only one community asset. The respondents who reported participating in at least one community asset reported participation in an average of two assets. On average, community asset participants had an EQ-5D score of 0.690 compared with 0.596 for non-participants. The difference in EQ-5D scores between participants and non-participants equalled 0.094 (95% CI 0.077 to –0.111; table 1).

**Table 1:** Characteristics by community asset participation status

Variable	Non-participant	Participant	Difference	95% CI
Health-related quality of life				
EQ5D Health Utility Index	0.596	0.690	0.094	(0.0767 to 0.1107)
Healthcare usage (months)				
GP visits in 6	3.252	2.927	−0.326	(−0.5191 to −0.1320)
Hospital outpatient visits in 6	2.456	2.159	−0.297	(−0.5180 to −0.0760)
Ambulance call-outs in 6	0.418	0.218	−0.200	(−0.3353 to −0.0637)
Visits to casualty in 6	0.556	0.439	−0.117	(−0.2040 to −0.0299)
Total cost (£) of healthcare in 6	544.77	447.83	−96.94	(−161.25 to −32.64)
Demographic characteristics (years)				
Female participants	0.505	0.521	0.015	(−0.0169 to 0.0477)
Aged 65–69	0.296	0.289	−0.006	(−0.0358 to 0.0229)
Aged 70–74	0.257	0.275	0.017	(−0.0115 to 0.0456)
Aged 75–79	0.202	0.223	0.021	(−0.0053 to 0.0475)
Aged 80–84	0.136	0.124	−0.012	(−0.0339 to 0.0096)
Aged 85 and over	0.109	0.0894	−0.020	(−0.0390 to −0.0003)
Education				
School-level qualifications	0.153	0.317	0.164	(0.1373 to 0.1910)
College-level qualifications	0.0390	0.122	0.083	(0.0658 to 0.1005)
University-level qualifications	0.0423	0.0861	0.044	(0.0286 to 0.0602)
NVQ and trade qualifications	0.211	0.258	0.047	(0.0196 to 0.0743)
Professional qualifications	0.136	0.239	0.102	(0.0771 to 0.1272)
Living arrangements				
Lives alone	0.351	0.354	0.002	(−0.0285 to 0.0333)
Lives with spouse	0.568	0.591	0.023	(−0.0090 to 0.0549)
Lives with other	0.131	0.103	−0.028	(−0.0488 to −0.0074)
Health conditions				
Asthma	0.152	0.139	−0.013	(−0.0358 to 0.0099)
Cancer	0.0780	0.0795	0.002	(−0.0156 to 0.0191)
Back pain/sciatica	0.342	0.304	−0.037	(−0.0676 to −0.0072)
Bronchitis/COPD	0.170	0.115	−0.056	(−0.0784 to −0.0333)
Kidney disease	0.0542	0.0345	−0.021	(−0.0344 to −0.0077)
Colon/irritable bowel	0.141	0.157	0.016	(−0.0071 to 0.0388)
Congestive heart failure	0.0618	0.0422	−0.019	(−0.0336 to −0.0050)
Diabetes	0.234	0.201	−0.034	(−0.0607 to −0.0075)
Hard of hearing	0.412	0.409	−0.001	(−0.0331 to 0.0304)
Heart disease/angina	0.247	0.221	−0.024	(−0.0514 to 0.0032)
High blood pressure	0.532	0.530	−0.003	(−0.0356 to 0.0289)
High cholesterol	0.454	0.441	−0.013	(−0.0454 to 0.0188)
Osteoarthritis	0.304	0.317	0.015	(−0.0154 to 0.0444)
Osteoporosis	0.141	0.116	−0.026	(−0.0479 to −0.0047)
Overweight	0.404	0.405	0.001	(−0.0304 to 0.0329)
Poor circulation in legs	0.409	0.328	−0.079	(−0.1101 to −0.0480)
Rheumatoid arthritis	0.169	0.110	−0.058	(−0.0804 to −0.0358)
Rheumatic disease	0.0347	0.0302	−0.004	(−0.0158 to 0.0070)
Stomach problem/ulcer/etc.	0.241	0.249	0.009	(−0.0186 to 0.0369)
Stroke	0.0726	0.0680	−0.005	(−0.0214 to 0.0116)
Thyroid disorder	0.112	0.131	0.019	(−0.0025 to 0.0398)
Problems with vision	0.470	0.446	−0.022	(−0.0545 to 0.0098)
Other health condition	0.0856	0.0965	0.011	(−0.0080 to 0.0292)
Sample size	1857	1829		

COPD, chronic obstructive pulmonary disease; GP, general practitioner; NVQ, National Vocational Qualification.

There were few differences between the two groups in other characteristics including the prevalence of long-term conditions. An exception was the level of education; 9% of community asset participants had university qualifications compared with 4% of non-participants.

On average, community asset participants had visited a GP three times in the previous 6 months. This was 0.326 (95% CI −0.519 to −0.132) visits fewer than non-participants. Community asset participants reported an average of 2.2 hospital outpatient appointments in the last 6 months, 0.297 (95% CI −0.518 to −0.076) visits fewer than non-participants. Participants also reported fewer ambulance call-outs and casualty visits. The average healthcare costs over a 6-month period of community asset participants were £97 (95% CI −£161.25 to −£32.64) lower than non-participants (£448 vs £545).

### Multivariate analysis

When we controlled for sociodemographic characteristics, people who participated in community assets had EQ5D scores that were 0.081 (95% CI 0.064 to 0.098) higher than non-attenders (table 2; column 1). When we added in information on the presence of 23 specific health conditions (column (2)), this effect dropped to 0.063 (95% CI 0.048 to 0.077). Controlling for whether the conditions were limiting reduced the effect to 0.039 (95% CI 0.025 to 0.052; column (3)).

**Table 2:** Linear regression of health-related quality of life on community asset participation

Dependent variable:	EQ5D Health Utility Index		
Model specification	(1)	(2)	(3)
Community asset participation	0.0809***	0.0626***	0.0387***
	(0.064 to 0.098)	(0.048 to 0.077)	(0.025 to 0.052)
Female participants	−0.0381***	−0.0111	−0.00884
	(−0.056 to −0.021)	(−0.027 to 0.005)	(−0.022 to 0.005)
Aged 65–69 years	Reference category		
Aged 70–74 years	0.00277	−0.00151	−0.00793
	(−0.020 to 0.025)	(−0.020 to 0.017)	(−0.025 to 0.009)
Aged 75–79 years	−0.0180	−0.00695	−0.00853
	(−0.042 to 0.006)	(−0.027 to 0.013)	(−0.026 to 0.009)
Aged 80–84 years	−0.0405**	−0.0109	−0.0331**
	(−0.069 to −0.012)	(−0.035 to 0.013)	(−0.056 to −0.011)
Aged 85–98 years	−0.120***	−0.0680***	−0.0706***
	(−0.153 to −0.088)	(−0.098 to −0.038)	(−0.098 to −0.043)
Socioeconomic characteristics†	Yes	Yes	Yes
Health conditions‡	No	Yes	Limiting
Goodness of fit (adjusted R^2^)	0.075	0.361	0.461

Sample size is N=3686.

95% CIs in brackets.

**p<0.01; ***p<0.001.

†Educational qualifications and living arrangements. See data section for full details.

‡See Data section for details on the health conditions. Column 2 includes indicators to show whether or not an individual has any of the 23 conditions. Column 3 includes an indicator to show whether or not the conditions are limiting—defined as a response of 4 or 5 on the Bayliss score.

EQ5D, EuroQol-5D.

The estimated effect of community asset participation of 0.039 can be compared with the effects of a number of conditions which limited daily activity. The coefficient on having limiting back pain (or sciatica) was −0.167 (95% CI −0.195 to −0.140) and the coefficient on having limiting osteoarthritis was −0.157 (95% CI −0.185 to −0.132). These two conditions are the most frequently reported limiting conditions (13% of respondents). Not attending community assets had an effect comparable with one quarter of the size of having back pain or osteoarthritis. The effect of diabetes was −0.053 (95% CI −0.102 to −0.005), ∼ 1.5 times the effect of community asset participation.

As a post hoc analysis, we estimated the effect of each of the individual assets on EQ5D scores (see online supplementary table A2.1 in the appendix). We ran separate models for each asset, and conditioned on the same variables presented in column (3) of table 2. The largest effect, in terms of size and significance, is for sports clubs. The effects of participation in religious, other, social, educational, Women's Institute and parent–teacher groups are also statistically significant. Groups for the elderly have a positive, but insignificant, effect on EQ5D scores.

10.1136/bmjopen-2016-012374.supp1 supplementary appendices

As a second post hoc analysis, we modelled the effects of participation on individual dimensions of the EQ5D. We present this graphically in online supplementary figure A1.1 (available in the online supplementary appendix). Participation increases the probability of reporting the highest level for each of the five domains and decreases the probability of reporting the lowest four levels. The general pattern of results is similar across all five domains, with the greatest positive effect on usual activity, self-care and mobility, and the smallest effects on pain/discomfort and anxiety/depression.

People who participated in community assets had visited a GP around 0.222 (95% CI −0.418 to −0.026) times fewer over the previous 6 months than non-participants (table 3; column 1). However, this reduction became not statistically significant when we included information about health conditions. We observed a similar pattern for hospital outpatient appointments; community asset participants had fewer attendances but this reduction became not statistically significant when we controlled for the presence of health conditions.

**Table 3:** Linear regression of formal healthcare usage and costs on community asset participation and other characteristics

Dependent variable:	GP visits			Hospital visits			Costs (£) of healthcare usage		
	(1)	(2)	(3)	(4)	(5)	(6)	(7)	(8)	(9)
Community asset participation	−0.222*	−0.170	−0.0674	−0.293*	−0.210	−0.148	−75.17*	−53.32	−33.61
	(−0.418 to −0.026)	(−0.365 to 0.025)	(−0.259 to 0.124)	(−0.517 to −0.070)	(−0.428 to 0.008)	(−0.366 to 0.070)	(−141.43 to −8.90)	(−117.61 to 10.97)	(−97.10 to 29.88)
Socioeconomic characteristics†	Yes	Yes	Yes	Yes	Yes	Yes	Yes	Yes	Yes
Health conditions‡	No	Yes	Limiting	No	Yes	Limiting	No	Yes	Limiting
Goodness of fit (adjusted R^2^)	0.017	0.064	0.066	0.014	0.092	0.059	0.048	0.166	0.125

Sample size is N=3686.

95% CIs in brackets.

*p<0.05.

†Models also include gender, age, educational qualifications and living arrangements. See Data section for full details.

‡See Data section for details on the health conditions. Column 2 includes indicators to show whether or not an individual has any of the 23 conditions. Column 3 includes an indicator to show whether or not the conditions are limiting—defined as a response of 4 or 5 on the Bayliss score.

GP, general practitioner.

Individuals who participated in community assets used NHS resources worth £75.17 (95% CI −141.43 to −8.90) less than non-participants (table 3; column 7). However, as with the analysis of GP visits and outpatient attendances, this difference became not statistically significant when we added in information on underlying health conditions.

### Valuing the effects of participation in community assets

Using the current NICE threshold values of £20 000 to £30 000 per QALY, we estimated that the net benefits of community asset participation were £763 (95% CI 478.21 to 1047.75) to £1142 (95% CI 725.00 to 1557.91) per participant per year (table 4). Using the threshold value proposed by Claxton *et al*^27^, we gave a net benefit estimate of £496 per participant per year (95% CI 302.13 to 689.11).

**Table 4:** Linear regression of net benefits on community asset participation and other characteristics

Value of a QALY	£20 000 per QALY	£30 000 per QALY	£12 936 per QALY
Community asset participation	763.00***	1141.50***	495.60***
	(478.21 to 1047.75)	(725.00 to 1557.91)	(302.13 to 689.11)
	p<0.001	p<0.001	p<0.001
Goodness of fit (adjusted R^2^)	0.462	0.464	0.454

Sample size is N=3686.

95% CIs in brackets.

Models also include gender, age, socioeconomic characteristics and 23 indicators for presence of limiting long-term health conditions.

***p<0.001.

QALY, quality-adjusted life year.

## Discussion

### Statement of principal findings

Among individuals aged 65 years and over with long-term health conditions, we found that 50% of respondents had participated in at least one of 13 different types of community assets within the previous 6 months. Participants had similar demographic, socioeconomic and health characteristics as non-participants. Controlling for a range of characteristics, we found that participation in at least one community asset was associated with significantly higher HRQoL. Community asset participation was associated with lower usage of formal healthcare, though this association was not statistically significant when we controlled for sociodemographic characteristics and the presence of limiting long-term conditions. Combining the health benefits and reductions in healthcare costs, we found that community asset participation was associated with a net benefit of £763 per participant per year.

### Strengths and weaknesses of the study

Our analysis was based on a large data set collected from over 3600 older individuals who had one of 23 long-term health conditions and lived in a largely deprived area. This population group experiences compromised quality of life and makes substantial use of health and social care services. A major strength of this study is the opportunity to analyse such a large data set on this target population group. The response rate to the survey was similar to that of previous survey studies in similar populations.^28^ ^29^

The main limitation of this work is the reliance on cross-sectional data. We can show associations between community asset participation, HRQoL and costs, but we cannot assert causality. We find that HRQoL is better for community asset participants, but we cannot be sure whether better HRQoL predicts community asset participation, or if there is an unmeasured variable which is correlated with asset use and better HRQoL. An obvious contender variable is the prevalence of underlying health conditions. We showed that controlling for a wide range of 23 long-term health conditions reduced the differences in quality of life and costs between participants and non-participants but did not eliminate the difference in quality of life. We were also able to identify and control for the more severe of these conditions that limited daily activities and a significant difference in quality of life remained.

We have estimated all of our regressions using linear models. Given that the EQ5D follows a non-normal distribution, and is bound below by −0.594 and above by 1, it may be theoretically preferable to use other functional forms.^30^ However, there is evidence that Ordinary Least Squares (OLS) does just as well, if not, better than other estimators when analysing EQ5D scores.^30^ In robustness checks, we used other model specifications, but the results were qualitatively equivalent and the presentation and interpretation of the coefficients from linear models were more intuitive. A new value set for the EQ-5D-5L has been made available but is currently unpublished.^31^ We reran our analyses with this new value set (results not shown) and the magnitude of the effects were very similar.

An additional limitation is the definition of community asset participation. We treated individuals as participants if they ticked yes to participation in any of the groups in a specified list. This measure has not been formally validated, and we cannot be sure that it is comprehensive. There may be other community assets of relevance to health and quality of life beyond the groups explicitly listed in the questionnaire. We conducted secondary analyses to explore the effects of participation in each of the community assets separately, but would suggest caution in the interpretation of these disaggregated results. Discussions with user groups suggested that the same asset may be reported under different terms in the list. Despite its lack of discrimination between assets, the overall measure of asset use we used for the main analyses may be a more robust indicator of the effects of community asset participation.

This study used a complete case analysis. This may have introduced selection bias, but when we compared unadjusted averages of the variables available on the full sample as well as the estimation sample, the values were very similar.

The data were collected in a single city. This city is one of the most deprived areas in England and therefore represents an important target population for community-based assets development activity. However, the population is predominantly from white ethnic groups and further research in other populations would be necessary to assess the wider generalisability of the findings.

Finally, our usage data only include four categories of healthcare services. Owing to the need to reduce burden on respondents and to ensure an acceptable response rate, we limited our measures of healthcare usage to a small number of core categories, including primary care and hospital use. We do not have data on some potentially important services, such as physiotherapy, diagnostic services and dieticians, which may be impacted by asset use. However, the services that we do include constitute over 90% of total healthcare spending.^32^

### Strengths and weaknesses in relation to other studies, discussing particularly any differences in results

There have been several qualitative studies of community asset participation, but there have been very few quantitative studies of the effect of community asset participation on health and care usage. Like Steffens *et al,*^14^ we found that community asset participation improved health. Unlike the Steffens *et al* study, we were unable to follow individuals over time, but we used a larger sample of individuals with more information on health and healthcare usage. We used this information to calculate the net societal benefits expected from participation in community assets.

Unlike a study by Gleibs *et al*,^33^ we did not find a significant reduction in GP visits. However, their intervention was a much more targeted form of social interaction, compared with the very broad notion of a community asset considered here. Variation in patterns of associations between studies highlights the need for further work to understand the relationships and the factors (related to care context, population or intervention) that might moderate those relationships.

### Meaning of the study: possible mechanisms and implications for clinicians or policymakers

Older people with long-term health conditions are a focus of considerable current policy attention and there is much interest in new ways of intervening to improve this population's reliance on public services. Our results suggest that participation in community asset is associated with significantly higher HRQoL, but the association with reduced healthcare usage and costs is not statistically significant. We found a substantial net benefit to society of community asset participation, but it is the value of the HRQoL gains that dominates the cost savings. Encouragement of community asset participation may improve quality of life but not reduce demand on public expenditure.

Several forms of support have shown beneficial effects on quality of life but limited impact on costs to the health and social care sector.^34^ Better support may increase individuals’ knowledge of available services, uncover unmet needs and encourage more proactive use of formal care services. Community assets may be a further example of increased support that does not provide a simple substitute for formal care services.

### Unanswered questions and future research

We have only explored the effects of any participation in community assets on quality of life and care costs. It is likely that the health benefits vary depending on the intensity and quality of interactions with community assets, which would be a useful avenue for future work. It is also likely that certain types of community assets are more beneficial than others and investment would be best directed at particular forms of community asset development.

Our analysis has focused on natural, cross-sectional variation in participation between individuals. Longitudinal analysis would be required to understand whether the beneficial effects of community asset participation increase or decrease over time. One intervention that aims to help people build the skills to manage their social group interaction and therefore to improve their social identity has been shown to improve mental health, well-being and social connectedness outcomes.^35^ Further, experimental evidence on the effects of encouraging and stimulating greater community asset participation is now required to support future investment decisions.

## References

[1] Cabinet Office. Building the big society—publications—GOV.UK (cited 25 November 2015). https://www.gov.uk/government/publications/building-the-big-society

[2] McLean J Asset based approaches for health improvement: redressing the balance. UK: Glasgow Centre for Population Health, 2011.

[3] Marmot M, Friel S, Bell R Closing the gap in a generation: health equity through action on the social determinants of health. Lancet 2008;372:1661–9. 10.1016/S0140-6736(08)61690-6 18994664

[4] Liu LJ, Guo Q Loneliness and health-related quality of life for the empty nest elderly in the rural area of a mountainous county in China. Qual Life Res 2007;16:1275–80. 10.1007/s11136-007-9250-0 17703375

[5] Loneliness and health: potential mechanisms: psychosomatic medicine [internet]. Lww (cited 27 November 2015). http://journals.lww.com/psychosomaticmedicine/Fulltext/2002/05000/Loneliness_and_Health__Potential_Mechanisms.5.aspx 10.1097/00006842-200205000-00005 12021415

[6] Foot J, Hopkins T A glass half-full: how an asset approach can improve community health and well-being. 2010 (cited 25 November 2015). (Great Britain Improvement and Development Agency). http://www.local.gov.uk/health/-/journal_content/56/10180/3511449/ARTICLE

[7] McKnight J, Kretzmann J Building communities from the inside out: a path toward finding and mobilizing a community's assets. Chicago: ACTA Publications, 1993.

[8] Morgan A, Ziglio E Revitalising the evidence base for public health: an assets model. Promot Educ 2007;14 (Suppl 2):17–22. 17685075 10.1177/10253823070140020701x

[9] Kretzmann J, McKnight JP Assets-based community development. Natl Civ Rev 1996;85:23–9. 10.1002/ncr.4100850405

[10] Mathie A, Cunningham G From clients to citizens: asset-based community development as a strategy for community-driven development. Dev Pract 2003;13:474–86. 10.1080/0961452032000125857

[11] Whiting L, Kendall S, Wills W An asset-based approach: an alternative health promotion strategy? Community Pract 2012;85:25–8.23029774

[12] Dobrof J, Heyman JC, Greenberg RM Building on community assets to improve palliative and end-of-life care. J Soc Work End Life Palliat Care 2011;7:5–13. 10.1080/15524256.2011.548044 21391075

[13] Haslam C, Cruwys T, Haslam SA ‘The we's have it’: evidence for the distinctive benefits of group engagement in enhancing cognitive health in aging. Soc Sci Med 2014;120:57–66. 10.1016/j.socscimed.2014.08.037 25222136

[14] Steffens NK, Cruwys T, Haslam C Social group memberships in retirement are associated with reduced risk of premature death: evidence from a longitudinal cohort study. BMJ Open 2016;6:e010164 10.1136/bmjopen-2015-010164 PMC4762089 26883239

[15] Cruwys T, Alexander Haslam S, Dingle GA Feeling connected again: interventions that increase social identification reduce depression symptoms in community and clinical settings. J Affect Disord 2014;159:139–46. 10.1016/j.jad.2014.02.019 24679402

[16] Cruwys T, Dingle GA, Haslam C Social group memberships protect against future depression, alleviate depression symptoms and prevent depression relapse. Soc Sci Med 2013;98:179–86. 10.1016/j.socscimed.2013.09.013 24331897

[17] Brooks R EuroQol: the current state of play. Health Policy 1996;37:53–72. 10.1016/0168-8510(96)00822-6 10158943

[18] Guide to the methods of technology appraisal 2013 | the-reference-case | guidance and guidelines | NICE. (cited 10 December 2015). https://www.nice.org.uk/article/pmg9/chapter/the-reference-case

[19] van Hout B, Janssen MF, Feng YS Interim scoring for the EQ-5D-5L: mapping the EQ-5D-5L to EQ-5D-3L value sets. Value Health 2012;15:708–15. 10.1016/j.jval.2012.02.008 22867780

[20] NHS reference costs 2014 to 2015—Publications—GOV.UK (cited 16 March 2016). https://www.gov.uk/government/publications/nhs-reference-costs-2014-to-2015

[21] Curtis L, Burns A Unit costs of health & social care 2015. University of Kent, UK: Personal Social Services Research Unit, 2015 http://www.pssru.ac.uk/project-pages/unit-costs/2015/index.php?file=full

[22] Madhavi Bajekal SP (National C for SR. Social capital and social exclusion: development of a condensed module for the Health Survey for England. 2001 (cited 16 March 2016). http://webarchive.nationalarchives.gov.uk/+/www.dh.gov.uk/en/Publicationsandstatistics/Publications/PublicationsStatistics/DH_4078277

[23] Kennedy A, Bower P, Reeves D Implementation of self management support for long term conditions in routine primary care settings: cluster randomised controlled trial. BMJ 2013;346:f2882 10.1136/bmj.f2882 23670660 PMC3652644

[24] Bayliss EA, Ellis JL, Steiner JF Seniors’ self-reported multimorbidity captured biopsychosocial factors not incorporated into two other data-based morbidity measures. J Clin Epidemiol 2009;62:550–557.e1. 10.1016/j.jclinepi.2008.05.002 18757178 PMC2743235

[25] Stinnett AA, Mullahy J Net health benefits: a new framework for the analysis of uncertainty in cost-effectiveness analysis. Med Decis Making 1998;18(2 Suppl):S68–80. 10.1177/0272989X98018002S09 9566468

[26] Shah K, Praet C, Devlin N Is the aim of the English health care system to maximize QALYs? J Health Serv Res Policy 2012;17:157–63. 10.1258/jhsrp.2012.011098 22767891

[27] Claxton K, Martin S, Soares M Methods for the estimation of The National Institute for Health and Care Excellence cost-effectiveness threshold. Health Technol Assess 2015; 19:1–504. 10.3310/hta19140 PMC4781395 25692211

[28] Reeves D, Hann M, Rick J Care plans and care planning in the management of long-term conditions in the UK: a controlled prospective cohort study. Br J Gen Pract 2014;64:e568–75. 10.3399/bjgp14X681385 25179071 PMC4141614

[29] Kenning C, Coventry PA, Gibbons C Does patient experience of multimorbidity predict self-management and health outcomes in a prospective study in primary care? Fam Pract 2015;32:311–16. 10.1093/fampra/cmv002 25715962 PMC4445135

[30] Maheswaran H, Petrou S, Rees K Estimating EQ-5D utility values for major health behavioural risk factors in England. J Epidemiol Community Health 2013;67:172–80. 10.1136/jech-2012-201019 22844084

[31] Devlin N, Shah K, Feng Y Valuing Health-Related Quality of Life: An EQ-5D-5L Value Set for England. London, UK: Office of Health Economics, 2016 Jan (cited 30 March 2016). Report No.: 16/01. https://www.ohe.org/publications/valuing-health-related-quality-life-eq-5d-5l-value-set-england 10.1002/hec.3564 PMC6680214 28833869

[32] Resource allocation: weighted capitation formula—Publications—GOV.UK. (cited 1 August 2016). https:// http://www.gov.uk/government/publications/resource-allocation-weighted-capitation-formula

[33] Gleibs IH, Haslam C, Haslam SA Water clubs in residential care: Is it the water or the club that enhances health and well-being? Psychol Health 2011;26:1361–77. 10.1080/08870446.2010.529140 21598183

[34] Panagioti M, Richardson G, Small N Self-management support interventions to reduce health care utilisation without compromising outcomes: a systematic review and meta-analysis. BMC Health Serv Res 2014;14:356 10.1186/1472-6963-14-356 25164529 PMC4177163

[35] Haslam C, Cruwys T, Haslam SA Groups 4 health: evidence that a social-identity intervention that builds and strengthens social group membership improves mental health. J Affect Disord 2016;194:188–95. 10.1016/j.jad.2016.01.010 26828756

