# Supplementary material for: Associations of participation in community assets with health-related quality of life and healthcare usage: a cross-sectional study of older people in the community
Source: BMJ Open. 2017 Feb 9;7(2):e012374. doi: 10.1136/bmjopen-2016-012374 (PMC5306503; doi:10.1136/bmjopen-2016-012374)
Supplement: supplementary appendices [file bmjopen-2016-012374supp_appendices.pdf]

ONLINE APPENDIX

Appendix A1: Supplementary Figures

Figure A1.1: The effect of community asset participation on each of the EQ-5D-5L Domains

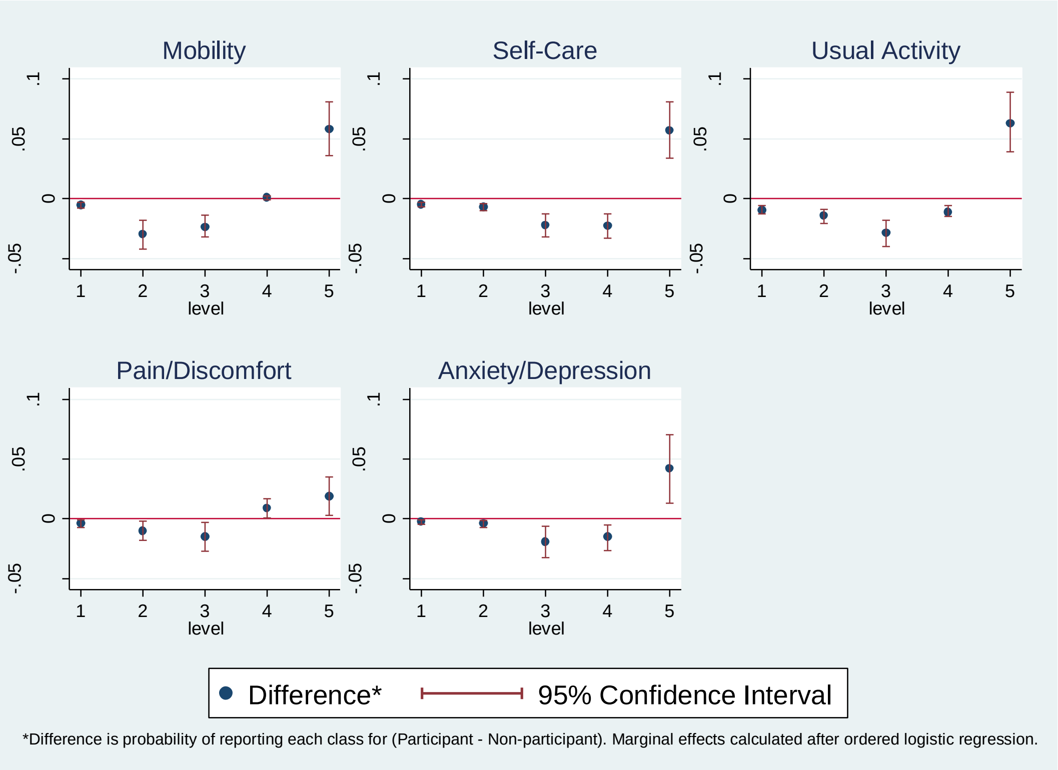

For each of the five domains, we ran separate ordered logistic regressions of the response category against the same set of control variables used in Table 2, column (3). We then obtain the marginal effects to establish the difference in the probability of reporting each level by community asset participation.

Appendix A2: Supplementary Tables

Table A2.1: Separate linear regressions of Health Related Quality of Life on participation in each type of community asset

Type of community asset	b	p-value	CI	Number (%) who partake
Religious	0.0226**	(0.008)	[0.006 to 0.039]	677 (18.35%)
Sports club	0.0628***	(0.000)	[0.049 to 0.077]	660 (17.89%)
Other group	0.0184*	(0.035)	[0.001 to 0.035]	558 (15.13%)
Social club	0.0188*	(0.027)	[0.002 to 0.036]	518 (14.04%)
Charity	0.00638	(0.528)	[-0.013 to 0.026]	477 (12.93%)
Group for elderly	0.00359	(0.749)	[-0.018 to 0.026]	421 (11.41%)
Education	0.0342**	(0.002)	[0.012 to 0.056]	255 (6.91%)
Women's institute	0.0412*	(0.012)	[0.009 to 0.073]	141 (3.82%)
Teacher or residents	0.0252	(0.118)	[-0.006 to 0.057]	100 (2.71%)
Environmental	-0.00887	(0.695)	[-0.053 to 0.035]	77 (2.09%)
Trade union	-0.00614	(0.808)	[-0.056 to 0.043]	48 (1.30%)
Parent-teacher	0.0523*	(0.024)	[0.007 to 0.098]	39 (1.06%)
Youth group	-0.0362	(0.368)	[-0.115 to 0.043]	33 (0.89%)

Each row is taken from a separate regression of EQ5D score on participation in the given asset.

Sample size is N=3,686 in all models.* p<0.05 ** p<0.01 ***p<0.001.

The same control variables are those reported in column (3) of Table 2.
